# Enhancing flotation separation of chalcopyrite and magnesium silicate minerals by surface synergism between PAAS and GA

**DOI:** 10.1038/s41598-021-85984-y

**Published:** 2021-03-18

**Authors:** Zhiqiang Chen, Yanhong Wang, Liqun Luo, Tiefeng Peng, Feng Guo, Mingyu Zheng

**Affiliations:** 1 Key Laboratory of Ministry of Education for Solid Waste Treatment and Resource Recycle, Southwest University of Science and Technology, Mianyang, 621010 Sichuan China; 2 School of Economics, Management & Law, University of South China, Hengyang, 421001 China; 3 The School of Minerals Processing and Bioengineering, Central South University, Changsha, China; 4 College of Resources and Environmental Engineering, Wuhan University of Technology, Wuhan, 430070 China

**Keywords:** Chemistry, Engineering

## Abstract

Separation effects of sodium polyacrylate (PAAS) and gum Arabic (GA) on flotation of chalcopyrite and magnesium silicate minerals using potassium butyl xanthate (PBX) as collector were investigated by micro-flotation experiments, zeta potential, Infrared spectral (IR), SEM–EDS, XPS analysis and copper sulphide ore beneficiation test. The micro-flotation experiments and zeta potential measurements showed that combined depressant consisting of PAAS and GA could efficiently reduce the recoveries of mixed minerals of serpentine and talc more than 25%, while that of chalcopyrite remained above 70% at pH 9.2. Infrared spectral (IR), SEM–EDS and XPS analysis showed that PAAS chemically reacted with Mg on the surface of serpentine, while GA adsorbed on talc surface mainly via physical interaction and hydrogen bond may also play a role. Surface synergism between PAAS and GA was investigated by turbidity test and its depression mechanism was proposed. The technology feasibility of using PAAS and GA to improve the copper sulphide ore flotation performance was verified through artificial mixed ore flotation and laboratory closed-flotation operation.

## Introduction

Most of the copper-nickel metals at present is obtained from copper-nickel sulphide ores, and the on-going development of industrial fields results in a steadily increasing demand for copper-nickel metals, but the development of industry is limited by the low recovery and low grade of Copper-Nickel Sulfide Ores^1,2^. Every year, a large number of Copper-Nickel Sulfide Ores imports to meet domestic demand, so the efficient utilization of copper-nickel resources is vital to the rapid development of country's economic. In order to meet the increasing demand for copper-nickel metals in China, a large number of minerals have been exploited, which makes the reserve of copper-nickel resources in China decrease significantly^3^.

The metal minerals in Copper-Nickel Sulfide Ores are mainly pentlandite, chalcopyrite and magnetite, and a large number of magnesium silicate minerals, such as serpentine, talc and so on. The composition of magnesium silicate minerals is complex, coexisted in an ore, and easily broken, forming a fine-grained mineral, which seriously affects the recovery of copper concentrate^4,5^. Serpentine is a hydrophilic mineral with poor natural floatability. After grinding, its surface is positively charged, so it is easy to cover the surface of negatively charged copper sulfide ore surface and prevent copper sulfide ore from reacting with collectors^6,7^. Talc is a hydrophobic mineral with excellent natural floatability. It will enter the concentrate product along with the foam during the flotation process, affecting the concentrate grade^8–11^. In the flotation process of Copper-Nickel Sulfide Ores, reducing the content of MgO in the concentrate is a technical goal of ore beneficiation.

Magnesium silicate minerals and metal sulfide minerals coexist in an ore, which increases the difficulty of flotation separation. At the same time, serpentine and talc are different types of magnesium silicate minerals whose surface properties and flotation behavior are different^12–14^. Separation scheme is different from that of sulfide ores, a large number of studies have shown that Carboxymethyl cellulose (CMC) is a common modifier for Copper-Nickel Sulfide Ores to reduce MgO content in concentrate^12,13,15,16^. As for the talc type Copper-Nickel Sulfide Ores, the talc entrained in flotation concentrate with foam will affect the grade of copper sulfide ore, so gum Arabic (GA) is a good depressant to separate copper sulfide ore and talc^4,8,17^. Although much works have been done on magnesium silicate minerals by many scholars, the poor solubility of many depressants normally results in large solution volume additions and directly affects the flotation recovery^15,18,19^.

Depressant is a good candidate to prevent magnesium silicate minerals from floating, many scholars are seeking an efficient and clean magnesium silicate minerals depressant^12,20–23^. Chen et al.^24^ showed that tetrasodium iminodisuccinate (IDS) can effectively eliminate the adverse effect of serpentine on the flotation of pyrite, suggesting that tetrasodium iminodisuccinate (IDS) can chemically react with the Mg sites on the surface of serpentine and form a soluble complex compounds, which causes potential displacement on the surface of serpentine. At the same time, IDS with negative charge adsorbed on the surface of serpentine effectively reduced the surface potential of serpentine, which removes serpentine from the pyrite surface. Zhao et al.^20^ showed that galactomannan could effectively depress the flotation of serpentine, but it had no obvious depression effect on the flotation of pentlandite. Feng et al.^25^ used combined depressant consisting of acidified water glass and locust bean gum to depress the serpentine and talc mixed minerals, and finally a copper concentrate with grade of 20.30% and recovery of 75.80% was achieved. The results show that acidified water glass can effectively disperse the fine serpentine particles from the surface of talc, and the adverse effect of talc on the recovery of sulfide ore can be eliminated by adding locust bean gum^25,26^. Previous studies showed many scholars mainly focus on single depressant, and there is a lack of report on combined depressant of magnesium silicate minerals.

In this study, the basic idea and mechanism of synchronous inhibition of magnesium silicate minerals by combined depressant consisting of PAAS and GA were studied. Sodium polyacrylate (PAAS) is a kind of water-soluble polymer with a high molecular weight^27^. There are only a few studies and applications have been reported in oxidizing mineral processing. PAAS can be decomposed into low-molecular-weight ions (Na^+^) and polymer ions in aqueous solution, the —COO— group may chemically react with Mg^2+^ on the surface of serpentine. Therefore, PAAS can be used as an efficient dispersant of serpentine in copper-nickel sulphide ores flotation system. Gum Arabic (GA) is derived from the trunk exudate of GA legume, and its main components are high molecular polysaccharides, calcium, magnesium and potassium salts, including gum aldose, galactose and glucuronic acid. It is found that it has a good depression effect on talc^28^. Therefore, focusing on the surface synergism of PAAS and GA on magnesium silicate minerals and mechanism will provide theoretical basis for the effective separation of chalcopyrite and magnesium silicate minerals in the future.

## Experimental

### Samples and reagents

**Figure 1:**
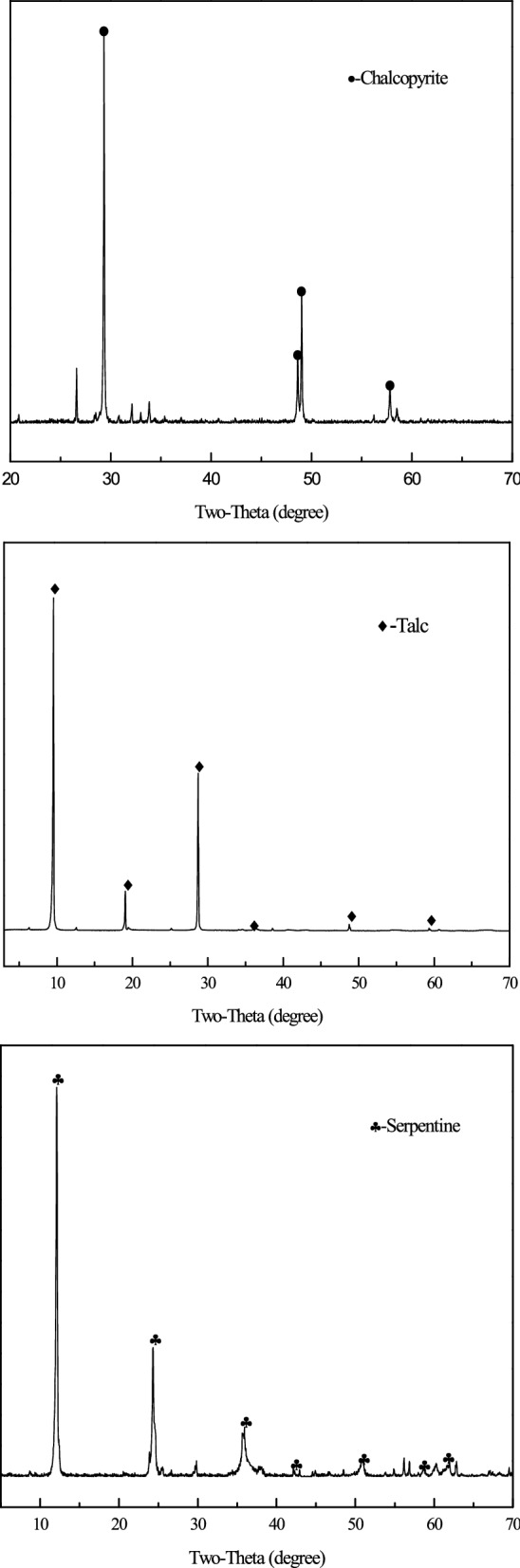
XRD patterns of the chalcopyrite, talc and serpentine.

Pure mineral sample of chalcopyrite was obtained from Lijiang, Yunnan Province, talc and serpentine were obtained from Shijiazhuang, Hebei Province. XRD and XRF analysis of the sample are shown in Fig. 1 and Table 1, respectively. Test samples were carefully selected, crushed, ground in an agate mortar, and sieved to obtain pure minerals with a particle size of -74 + 38 μm for flotation experiments. Then, the test sample was ground to -2 μm for further test, pure mineral samples were dried in a vacuum dryer at 35 °C to prevent oxidation, and stored in a sealed glass bottle. The Copper-Nickel Sulfide Ores used in this study were obtained from Xinjiang, China. The contents of these samples were 96.23% for chalcopyrite, 90.59% for serpentine and 94.44% for talc, respectively. The main chemical composition and mineral content of the ore samples are shown in Tables 2 and 3, respectively. Tables 1 and 2 are the results from XRF, Table 3 are the results from MLA analysis.

**Table 1:** Chemical compositions of chalcopyrite, serpentine and talc (mass fraction, %) by XRF analysis.

Compositions	Fe	Cu	S	MgO	SiO	Al_2_O_3_	CaO	Others
Chalcopyrite	28.15	34.20	33.88	–	3.77	–	–	–
Serpentine	2.80	–	–	36.82	50.33	0.64	4.35	5.06
Talc	0.70	–	–	31.39	63.05	–	–	4.86

**Table 2:** Contents of main elements by chemical analysis for Copper-Nickel Sulfide Ores (mass fraction, %).

Contents	Cu	Ni	Fe	S	Al_2_O_3_	K_2_O	TiO_2_	SiO_2_
Count	0.30	0.42	10.50	1.47	6.19	0.36	0.36	50.27
Contents	MnO	P_2_O_5_	Cl	Co	SrO	Cr_2_O_3_	CaO	MgO
Count	0.18	0.11	0.08	0.03	0.02	0.33	4.56	17.92

**Table 3:** Composition and contents of main minerals for Copper-Nickel Sulfide Ores (mass fraction, %).

Mineral	Pentlandite	Chalcopyrite	Pyrrhotite	Magnetite	Picotite	Ilmenite
Fraction	1.1	0.82	2.5	0.77	0.39	0.52
Mineral	Olivine	Serpentine	Talc	Biotite	Amphibole	Others
Fraction	5.3	16.1	53.2	10.7	7.8	0.8

Industrial grade potassium butyl xanthate (PBX) (collector, 92% purity) was obtained from the Chemical Factory of Wuhan, China. Pine oil(forther, 95% purity) was received from Shanghai Gaote Chemical Co., Ltd. Analytical grade sodium hydroxide and hydrochloric acid (NaOH and HCl, Hunan Zhuzhou Flotation Reagent Co., Ltd, China) were used as pH modifiers. Sodium polyacrylate (PAAS) and gum Arabic (GA) were purchased from Aladdin Reagent Co., Ltd. The reagents used in this study were analytical grade, and deionized water was used in all tests.

### Micro-flotation experiments

Micro-flotation test was performed on XFG (Changchun, China) flotation machine with impeller speed of 1700 r/min. The purified mineral particles (2.0 g) were placed in a plexiglass cell, and 35 mL of deionized water was injected into it. The pH of the suspension was adjusted with HCl or NaOH for 2 min, the required amount of depressant (PAAS and GA) and collector Potassium butyl xanthate (PBX) were sequentially added and stirred for 3 min. Finally, the pine oil was added to the cell with 1 min conditioning time. The pH of the suspension was measured and recorded before flotation. The flotation product was floated for 4 min and the recovery was calculated according to the dry weight of the product. The experimental results were measured three times under the same test condition, and the mean value was taken as the final result. Meanwhile, the standard deviation was calculated and presented as the error bar.

### Zeta potential measurements

Zeta potential^24,29,30^ was measured using ZETASIZER nano Zs90 series (Malvern Instrument Company, UK) to explore the interaction mechanism between depressants (PAAS and GA) and mineral particles. During the measurement, the conductivity and pH of suspension were continuously monitored, and the test temperature was kept at 25 ± 1 °C. Pure mineral particles were ground to − 2 μm by agate mortar, and the suspension for measurement was prepared by adding 30 mg mineral samples into 40 mL of the KNO_3_ background electrolyte solution with a concentration of 1 × 10^−3^ mol/L. The prepared suspension was magnetically stirred for 10 min, and the pH of the suspension was adjusted by HCl or NaOH. PAAS and GA (if needed) were added and conditioned for 10 min. Finally, the pH was recorded 5 min after solution settlement, and the supernatant was obtained for zeta potential measurement. Zeta potential was measured three times and its average value was taken as the final result, and the standard deviation was calculated and presented as an error bar.

### IR spectral analysis

The FT-IR instrument (Perkinelemer Instruments, USA) was used to obtain the FT-IR spectrum, which characterizes the interaction between depressants (PAAS and GA) and minerals. Spectral wavenumber range was 400 ~ 4000 cm^−1^, KBr tablet was applied. The purified mineral particles were first ground to -2 μm with an agate mortar, 0.5 g of the purified mineral particles was placed in a plexiglass cell with HCl or NaOH as a pH regulator. Then required reagent was added and conditioned for 30 min in the method described in the flotation experiment. Finally, the treated samples were washed three times with deionized water of the same pH and filtered. The samples to be measured by infrared spectroscopy should be dried in a vacuum dryer at 35 °C for 24 h.

### SEM–EDS

To determine the element components and produce images of serpentine and talc surface after treatment in different conditions, scanning electron microscopy coupled with energy dispersive spectroscopy (SEM–EDS) analysis were employed in this study. For SEM–EDS analysis, serpentine and talc samples were conditioned in the same process as in the flotation tests^24,29,30^.

### X-ray photoelectron spectroscopy analysis

The chemical composition of mineral samples was determined by X-ray energy spectrum^24,29^. 1.0 g sample was added to 50 mL deionized water. Subsequently, single depressant was added, after adjusting the pH, leave the suspension at 25 °C for 10 min. Then mineral samples were washed three times with deionized water of the same pH, filtered and dried in a 40 °C vacuum drying oven. XPS analysis was carried out using k-alpha 1063 X-ray spectrometer (Thermo Fisher, UK).

### Turbidity test

Turbidity tests were conducted to illustrate the coagulation and dispersion behavior of serpentine and talc. 0.1 g talc and 0.1 g serpentine were added to 80 mL distilled water. The regents were added and stirred for 5 min. The suspension was poured into a 100 mL volumetric flask, the pH of the suspension was measured and shaken up and down 20 times at the same time. Then it was allowed to stand for 3 min. Finally, 25 mL suspension was measured by WGZ—3. The dispersion of the supernatant liquor was characterized by its turbidity. A high turbidity value indicates that the sample is dispersed well^25^.

### Flotation of copper sulfide ore

**Figure 2:**
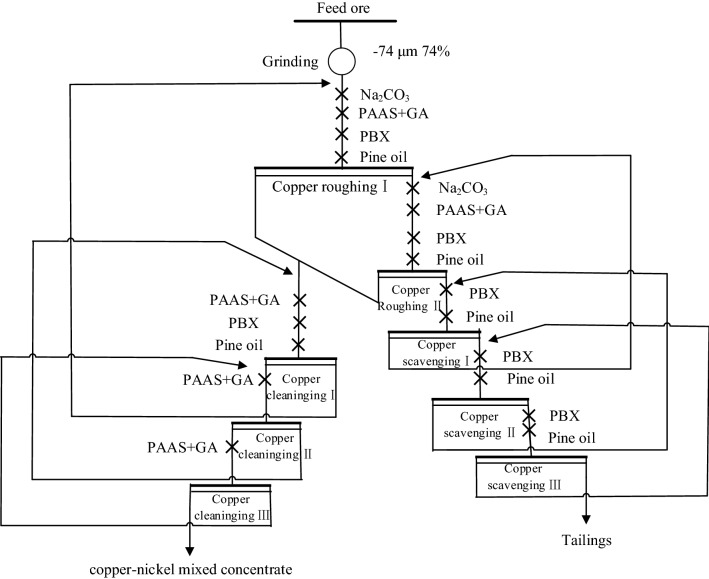
The flowsheet of laboratory closed-flotation operation for copper sulphide ore.

The test was carried out in the flotation cells (XFD-63) with 500 g sample. First, adjust the pH of the suspension with sodium carbonate for 2 min, add depressant and stir for 2 min, then add collector (PBX) and stir for 2 min, finally add foaming agent (Pine oil) and stir for 1 min. After 3 min of flotation, the concentrate and tailings were filtered and dried, the concentrate was repeated to form a closed-flotation separation test. Finally, mixed concentrate of copper-nickel was obtained. The flowsheet of laboratory closed-flotation operation for copper sulphide ore is shown in Fig. 2, flotation time was 3 min.

## Results and discussion

### Single mineral flotation results

**Figure 3:**
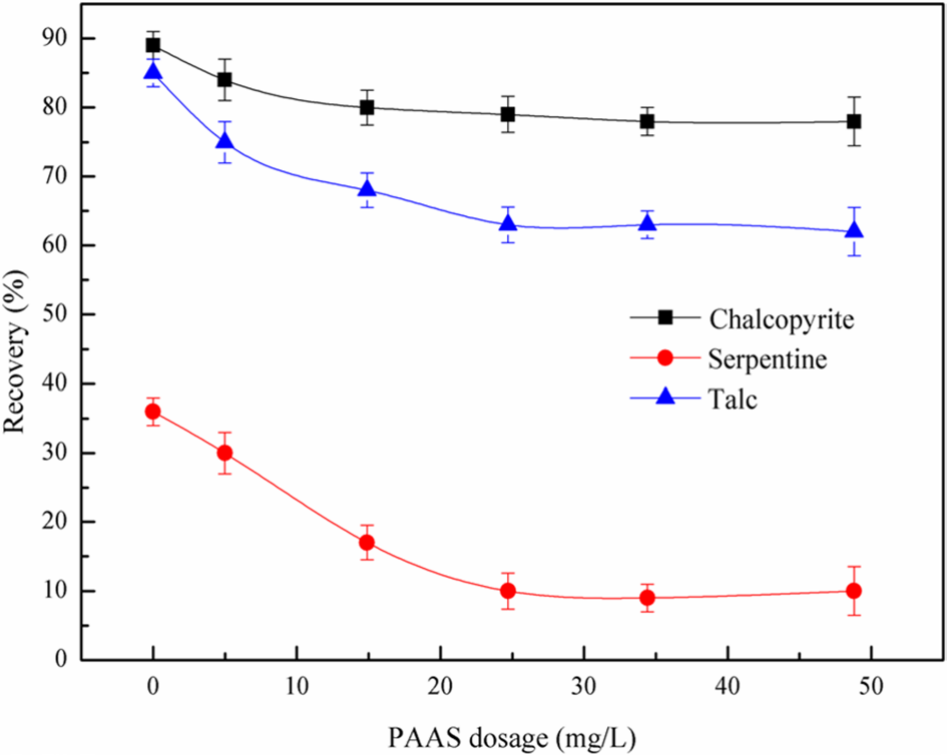
Effect of PAAS on flotation behavior of chalcopyrite, serpentine and talc at pH = 7, *c*(PBX) = 80 mg/L.

**Figure 4:**
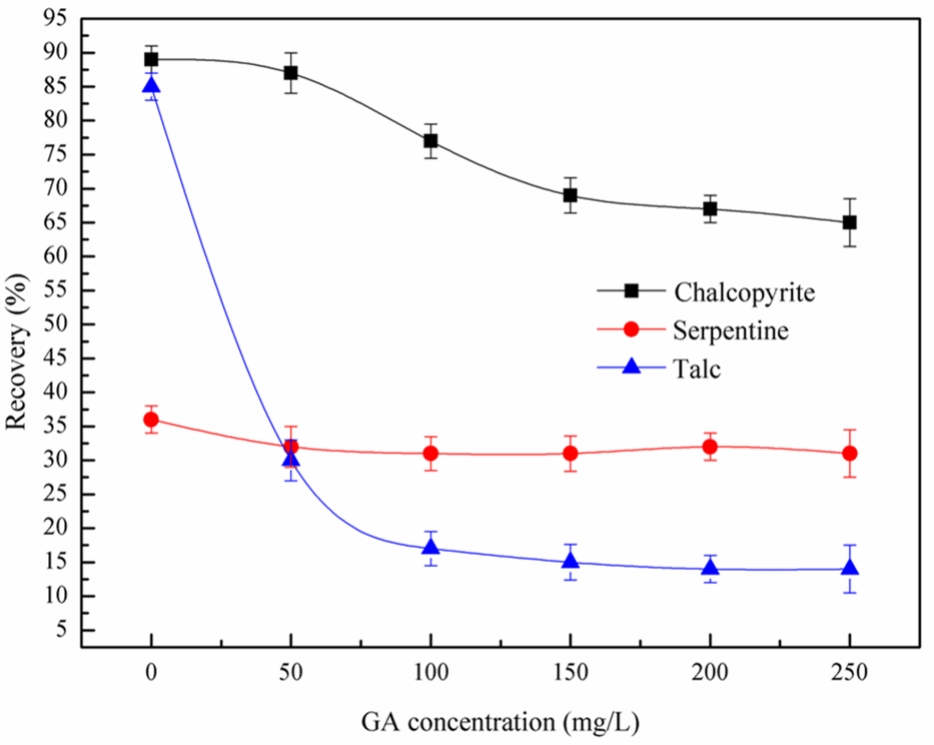
Effect of GA on flotation of chalcopyrite, serpentine and talc at pH = 7, *c*(PBX) = 80 mg/L.

**Figure 5:**
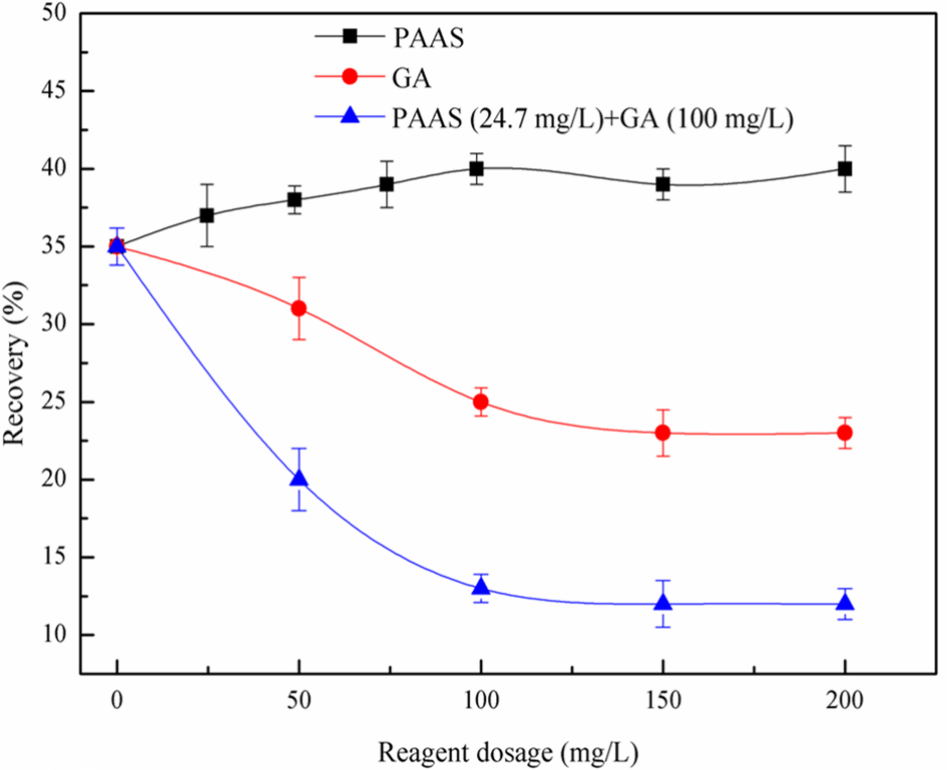
Effect of modifiers on flotation of serpentine and talc mixed minerals at pH = 7, *c*(PBX) = 80 mg/L, talc: − 150 + 37 μm, serpentine: − 10 μm.

A single mineral flotation experiment was carried out with PBX as collector, PAAS and GA as combined depressant. The effect of depressant dosage on the flotation recovery of chalcopyrite, serpentine and talc was studied. The results are shown in Figs. 3, 4 and 5.

Figure 3 showed the effect of PAAS dosage on the flotation behavior of chalcopyrite, serpentine and talc. The experiment was carried out at pH 7. It can be seen from Fig. 3 that the dispersant PAAS had significant influence on the flotation recovery of serpentine, but it had little depression effect on chalcopyrite and talc. When the PAAS dosage exceeds 24.7 mg/L, the recovery of serpentine decreased from 36 to 10%, which was the maximum depression effect of serpentine. Therefore, in the subsequent flotation test, 24.7 mg/L was considered as the optimal dosage of PAAS.

The effect of GA dosage on the flotation behavior of chalcopyrite, serpentine and talc was presented in Fig. 4. The experiment was carried out at pH 7. The recoveries of talc and chalcopyrite decreased with the increase of GA dosage, but GA had little depression effect on serpentine. Figure 4 showed that GA had a significant depression effect on talc, compared with talc, the depression effect of GA on chalcopyrite was weak. When the GA dosage exceeds 100 mg/L, the recoveries of chalcopyrite and talc were 77% and 17% respectively, which was the maximum depression effect of talc. Therefore, in the subsequent flotation test, 100 mg/L was considered as the optimal dosage of GA.

Figure 5 showed the effect of the dosage of regulators on the recovery of mixed minerals of serpentine and talc at pH 7. In the test, serpentine and talc were mixed at 1:1 (mass ratio), and the dosage of PAAS and GA were 24.7 mg/L and 100 mg/L, respectively. According to Fig. 5, when the dispersant PAAS was used alone, it had no significant depression effect on the mixed minerals of serpentine and talc; On the contrary, the recovery of the mixed ores increases with an increase in the dosage of PAAS. PAAS could remove fine serpentine from the surface of talc, which eliminated the adverse effect of serpentine on the flotation recovery of talc. It can be seen from Fig. 5 that the depression effect of GA alone on mixed minerals was weak. When PAAS of 24.7 mg/L was first added, the addition of GA had significant depression effect on the mixed minerals. PAAS could remove fine serpentine from the surface of talc, the addition of GA can effectively depress the flotation recovery of talc. Serpentine and talc can be synchroally depressed by PAAS and GA.

**Figure 6:**
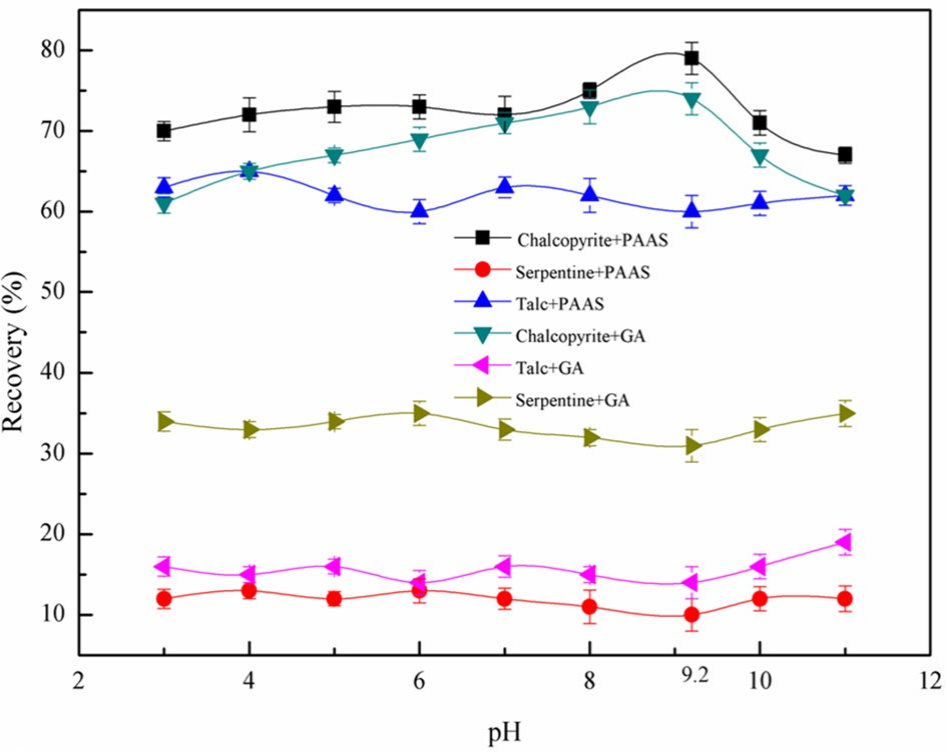
Effect of pH on flotation of minerals, *c*(PBX) = 80 mg/L, *c*(PAAS) = 24.7 mg/L, *c*(GA) = 100 mg/L.

The recoveries of chalcopyrite and magnesium silicate minerals as a function of the pH obtained using single-mineral flotation tests were shown in Fig. 6. The recoveries of chalcopyrite, serpentine and talc were 79%, 10% and 60%, respectively, when the pH was controlled at the vicinity of 9.2 with 80 mg/L PBX and 24.7 mg/L PAAS. The recoveries of chalcopyrite, serpentine and talc were 74%, 31% and 14%, respectively, when the pH was controlled at the vicinity of 9.2 with 80 mg/L PBX and 100 mg/L GA.

### Zeta potential results

**Figure 7:**
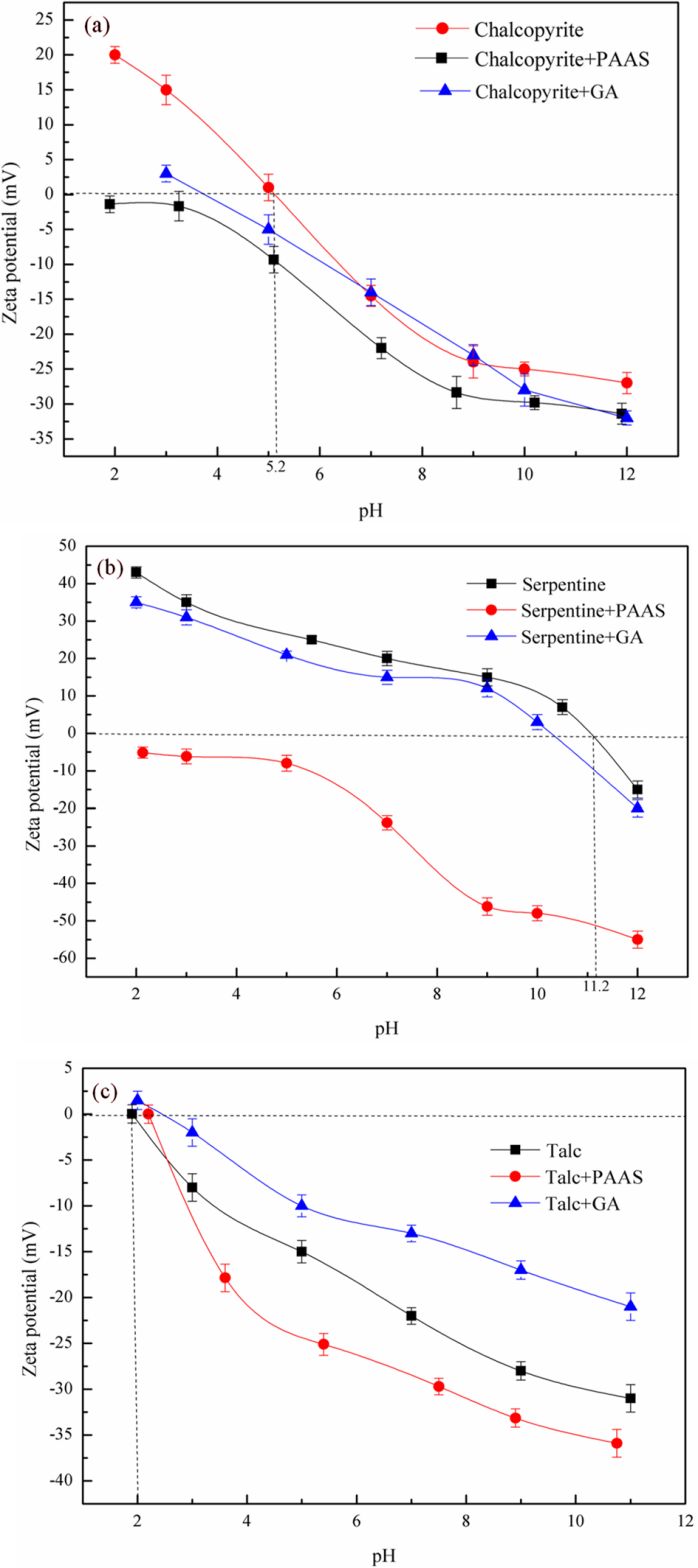
Zeta potentials of chalcopyrite (**a**), serpentine (**b**), and talc (**c**) at the different pH before and after reacting with PAAS and GA, c(PAAS) = 24.7 mg/L, c(GA) = 100 mg/L.

Adsorbats can change the surface properties of minerals and affect the flotation behavior of minerals. Zeta potential was one of the most useful tools for explaining the interactions between mineral surfaces and flotation reagents. Figure 7 showed the zeta potentials before and after the mineral treated with reagents in the pH range of 2–12. It can be seen from Fig. 7 that the IEP (Iso-Electric Point) of chalcopyrite, serpentine and talc were 5.2, 11.2 and 2.0^26,31–33^, respectively, which was consistent with the previous results^8,25,34^.

According to Fig. 7a, within the pH range of 2–12, the zeta potential of chalcopyrite decreased from 20 to − 27 mV. After treated with PAAS and GA, the zeta potential of chalcopyrite changed to be negative. Figure 7a also showed that PAAS and GA had little effect on the zeta potential of chalcopyrite, which indicated that PAAS and GA exhibited low sorption affinity on the surface of chalcopyrite^35^.

According to Fig. 7b, c, it can be seen that the zeta potential of serpentine (b), and talc (c) at the different pH before and after reacting with PAAS and GA. In the range of pH 8–11.2, the zeta potential of serpentine and talc were opposite, and there was significant electrostatic attraction between them. After the addition of dispersant PAAS, the surface potential of serpentine and talc changes, the zeta potentials of serpentine and talc were − 46.2 mV and − 33.75 mV at pH 9.0, so they repel each other through electrostatic interaction. It indicated that PAAS could make the zeta potential of serpentine and talc negatively change, which promote the dispersion of serpentine from the surface of talc, thus reducing the adverse influence of serpentine on the recovery of talc and the flotation results of Fig. 5 can be explained^13^. After the addition of GA, the zeta potential on the surface of talc changed significantly in the pH range of 2–12, indicating that GA had higher affinity on the surface of talc. Compared with talc, the zeta potential on the surface of serpentine before and after the addition of GA showed little change, indicating that GA exhibited low sorption affinity on the surface of serpentine, which was consistent with the above flotation results^36^.

In summary, by adding dispersant PAAS, the zeta potential on the serpentine surface changed from positive to negative and the chalcopyrite and talc surface became negatively charged in the pH range of 2–12. Therefore, the influence of serpentine slime on mineral flotation could be removed from talc and chalcopyrite surfaces by electrostatic repulsion. After the addition of depressant GA, both chalcopyrite and talc had negative surface charges under weakly alkaline pH flotation conditions, and they were separated due to electrostatic repulsion. At the same time, GA has a significant depression effect on the flotation recovery of talc, which could reduce the possibility of talc entering the concentrate with flotation froth, so the adverse effect of talc on the recovery of copper sulfide concentrate was reduced.

### IR results

The adsorption mechanism of PAAS and GA on the surface of chalcopyrite, serpentine and talc was studied by IR. For PAAS, its stretching bands of hydroxyl (–OH) appeared at 3422 cm^−1^, the stretching bands of –CH_3_ group emerged at 2926 cm^−1^, its stretching bands of carboxyl (–COO–) appeared at 1638 cm^−1^ and 1114 cm^−1^^37^. For GA, its stretching bands of hydroxyl (–OH) appeared at 3434 cm^−1^, the stretching bands of –CH_3_ or –CH_2_ group emerged at 2957 cm^−1^, its stretching bands of carboxyl (–COO–) appeared at 1626 cm^−1^, the bending vibration peak of –CO– group emerged at 1280 cm^−1^ and 1020 cm^−1^^38^.

**Figure 8:**
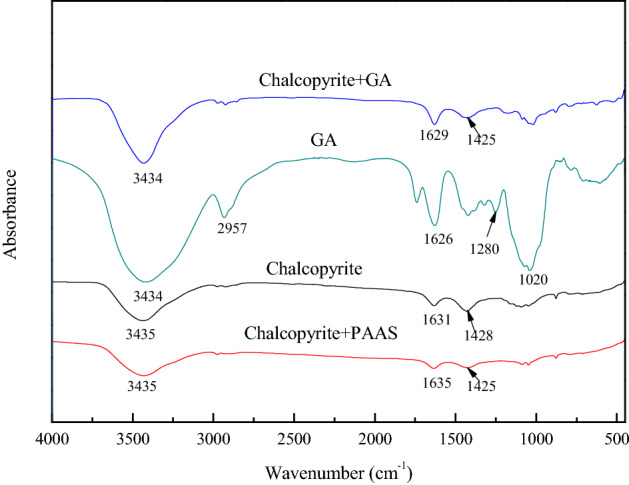
IR spectra of chalcopyrite in the absence and presence of flotation reagents, c(PBX) = 80 mg/L, c(PAAS) = 24.7 mg/L, c(GA) = 100 mg/L.

Figure 8 showed the IR spectra of chalcopyrite in the absence and presence of PAAS and GA. It can be seen that the impact of PAAS on chalcopyrite was negligible. After treatment with PAAS and GA, no new characteristic IR peaks of chalcopyrite were observed, which is consistent with the situation of chalcopyrite before and after PAAS and GA treatment in Fig. 7, indicating that the adsorption of PAAS and GA to chalcopyrite were weak.

**Figure 9:**
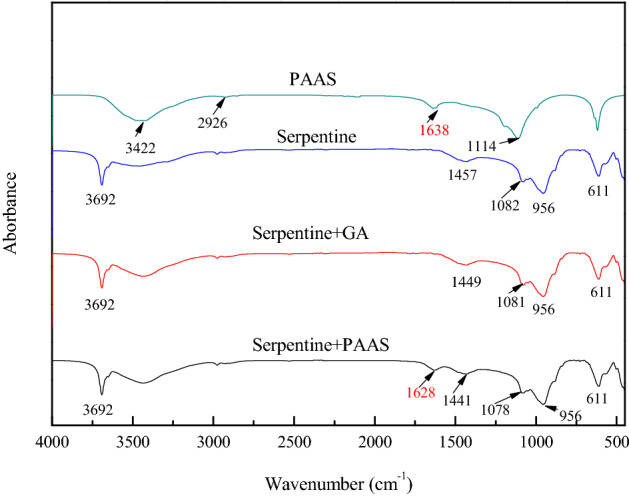
IR spectra of PAAS and serpentine conditioned in different reagents, c(PBX) = 80 mg/L, c(PAAS) = 24.7 mg/L, c(GA) = 100 mg/L.

The IR spectra of serpentine conditioned in different regents are shown in Fig. 9. For serpentine, its stretching bands of hydroxyl (–OH) appeared at 3692 cm^−1^. Other researchers also studied serpentine, the absorption at 1082 cm^−1^ was caused by the tensile vibration of Si–O, and the out of plane bending vibration of Mg-O occurred at 611 cm^−1^^16,18^. After the serpentine was treated with PAAS, a new absorption peak appeared at 1628 cm^−1^. Compared with the absorption peak at 1638 cm^−1^ in PAAS, it moved 10 cm^−1^ to the low frequency end, indicating that PAAS had chemical adsorption on the surface of serpentine.

**Figure 10:**
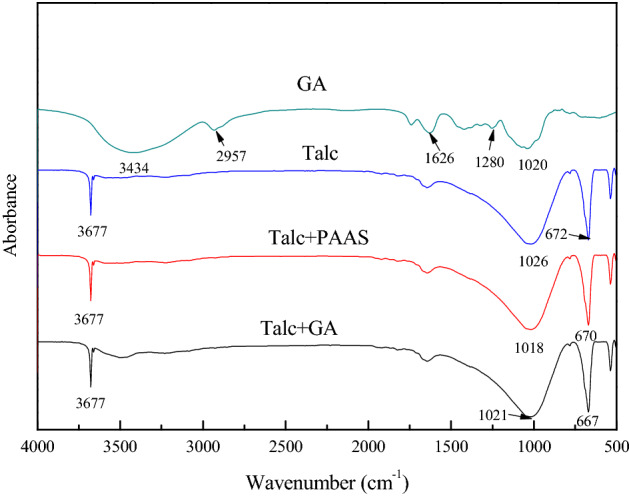
IR spectra of GA and talc conditioned in different reagents, c(PBX) = 80 mg/L, c(PAAS) = 24.7 mg/L, c(GA) = 100 mg/L.

Figure 10 showed IR spectra of talc conditioned in different regents. For bare talc, its stretching bands of hydroxyl (–OH) appeared at 3677 cm^−1^, the stretching bands of Si–O group emerged at 1026 cm^−1^, and the out of plane bending vibration of Mg-O occurred at 611 cm^−1^^39^. After the treatment of talc with PAAS and GA, no significant changes were observed in the infrared spectrum of talc, indicating that PAAS and GA did not chemically adsorb on the surface of talc. GA may physically react with the surface of the talc and hydrogen bond may also play a role.

### SEM–EDS analysis

**Figure 11:**
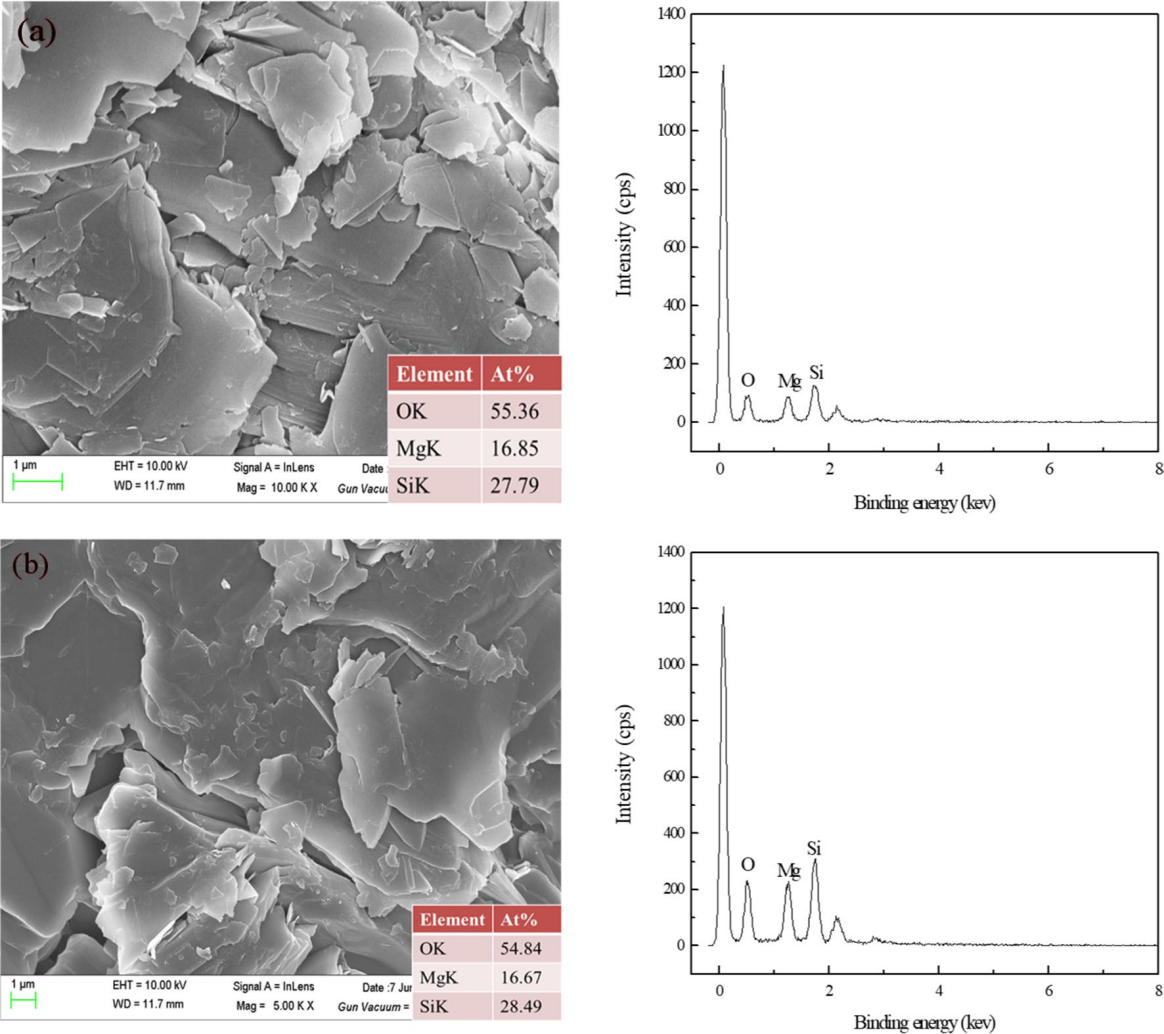
SEM–EDS analysis of talc samples: (**a**) talc; (**b**) talc treated with GA (GA = 100 mg/L).

SEM–EDS analysis results of talc before and after GA treatment were shown in Fig. 11. It can be seen from Fig. 11a that only O, Mg and Si were detected on the untreated talc surface, indicating that the talc was pure. After the treatment with GA (Fig. 11b), no new elements were detected and the surface morphology of talc had no changes, which indicated that GA did not adsorb on the surface of talc. It was further proved that GA physically reacted with the surface of the talc^40,41^.

**Figure 12:**
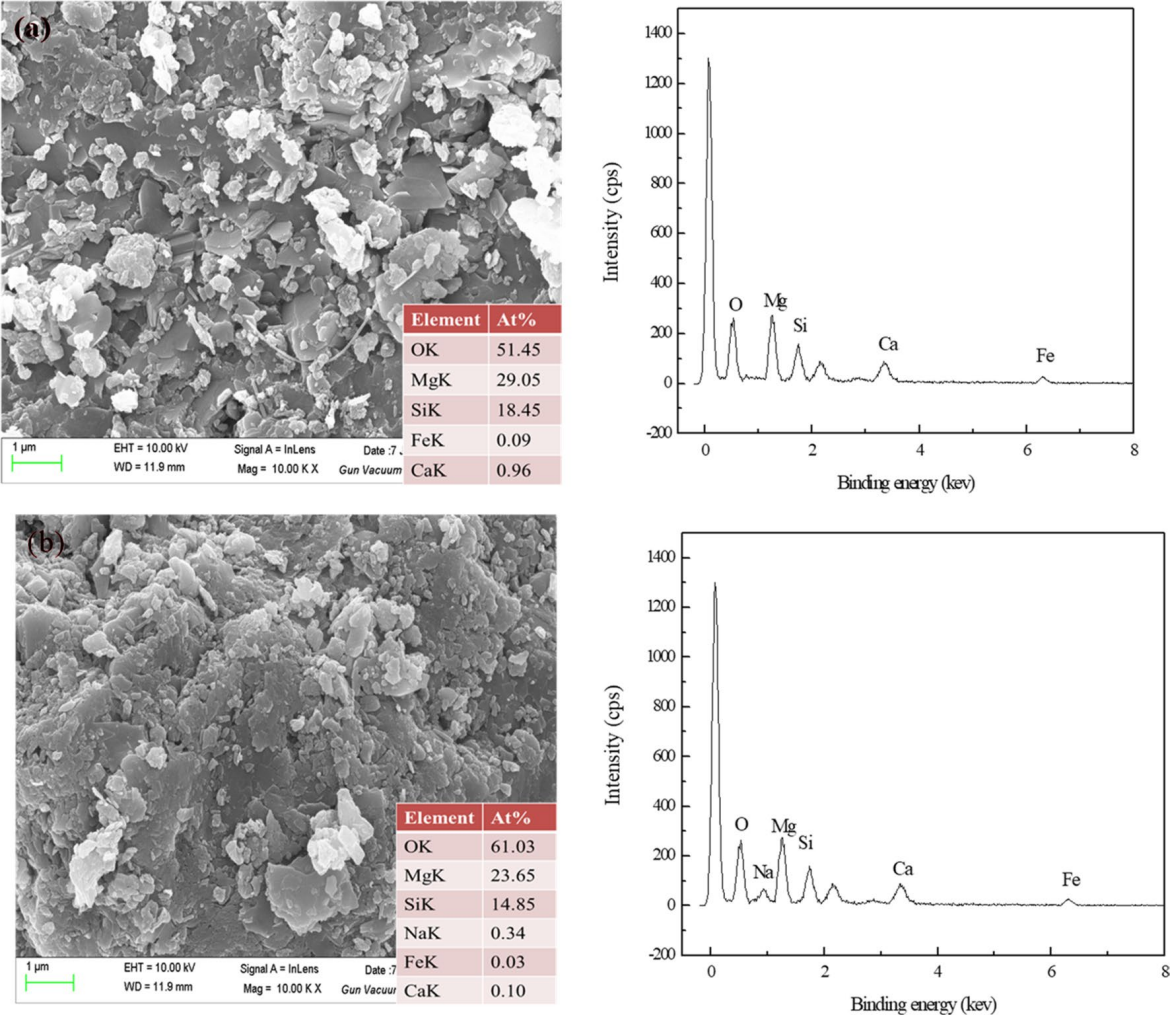
SEM–EDS analysis of serpentine samples: (**a**) serpentine; (**b**) serpentine treated with PAAS (PAAS = 24.7 mg/L).

SEM–EDS analysis results of serpentine before and after PAAS treatment were shown in Fig. 12. As shown in Fig. 12, the surface morphology of serpentine changed significantly after PAAS treatment. Meanwhile, for serpentine surface untreated with PAAS (Fig. 12a), only Mg, Ca, Fe, Si and O were detected, suggesting that serpentine was pure. After treatment with PAAS (Fig. 12b), 0.34% Na was detected and the relative atomic concentration of Mg decreased from 29.05 to 23.65%, which revealed the adsorption of PAAS onto serpentine surface and the dissolution of Mg from serpentine surface. The positive surface charge of serpentine is mainly attributed to the preferential dissolution of hydroxyl^42^. It may be that the carboxyl groups on PAAS chemically reacted with Mg on the serpentine surface, which accelerated the dissolution of Mg from the serpentine surface. PAAS dissolved in water has a negative charge. The adsorption of PAAS onto serpentine surface will reduce the surface potential of serpentine, which explains the test results in Fig. 7b.

**Figure 13:**
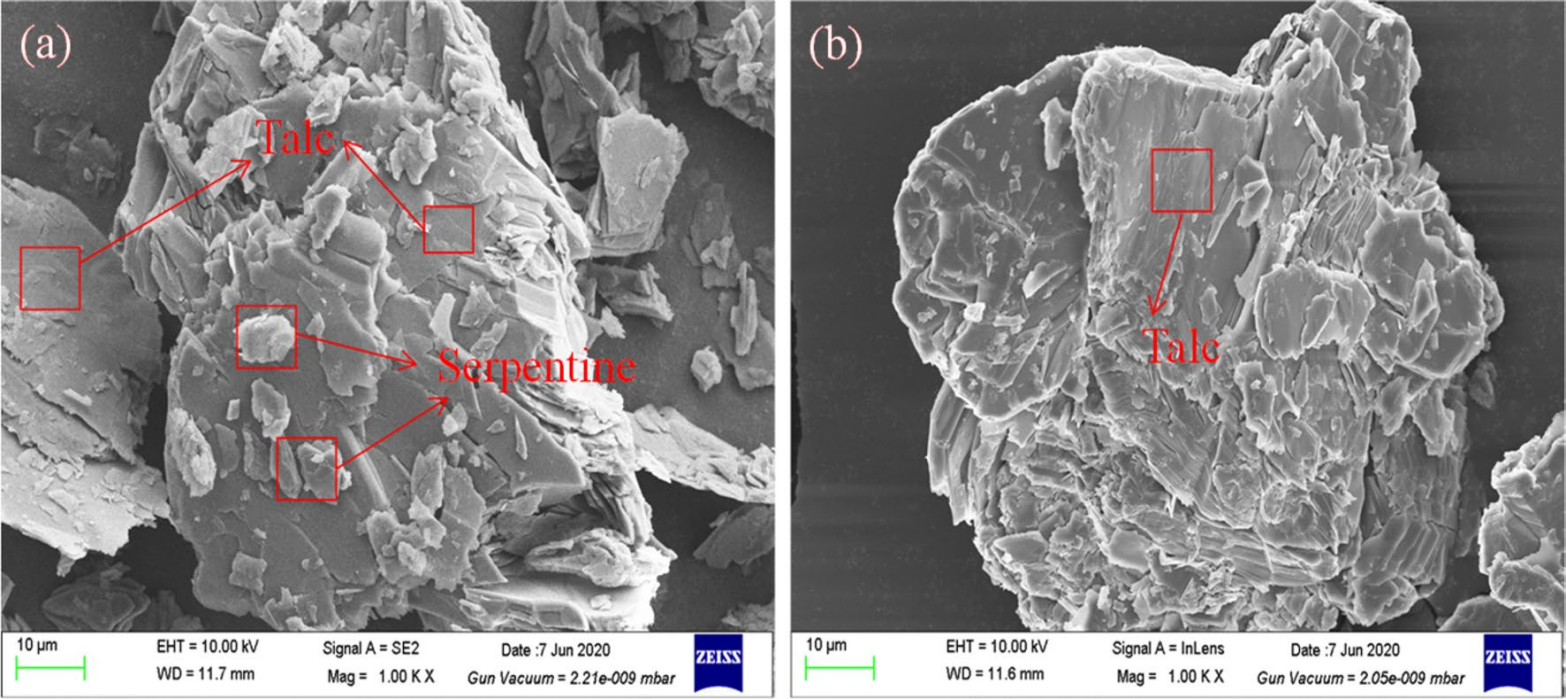
SEM analysis of mixed minerals of serpentine and talc: (**a**) mixed minerals of serpentine and talc; (**b**) mixed minerals of serpentine and talc with PAAS (PAAS = 24.7 mg/L).

In order to verify whether there is heterogeneous condensation between serpentine and talc, the dispersing effect of PAAS on serpentine was observed by SEM. The test results are shown in Fig. 13. In Fig. 13a, the dark part is the talc surface, and the bright part is the fine serpentine particles, which indicates that the fine serpentine particles are adsorbed on the talc surface by heterogeneous condensation. It can be seen from Fig. 13b that the fine serpentine particles on the talc surface are effectively reduced after the addition of PAAS, indicating that PAAS effectively weakens the heterogeneous condensation and makes the serpentine particles covered on the talc surface fall off.

### XPS analysis

To further explain the mechanism of action between PAAS and serpentine, the relative contents of elements on the serpentine surface in the absence and presence of PAAS, as measured by XPS, are listed in Table 4. After serpentine was treated by PAAS, the relative content of O reduced to 1.32%. After treated with PAAS, the content of O and Mg decreased by 1.32% and 1.29%, respectively, but the content of Si increased by 0.79%.

**Table 4:** Relative contents of elements on the serpentine surface.

Sample	Surface atomic composition (%)			
	C 1s	O 1s	Mg 1s	Si 2p
Serpentine	12.54	54.76	21.10	12.20
Serpentine + PAAS	13.76	53.44	19.81	12.99

**Figure 14:**
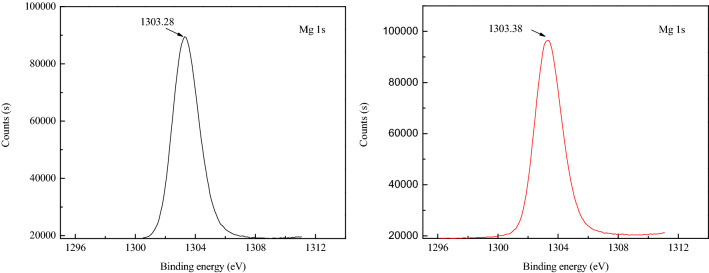
XPS spectra Mg (1s) of Serpentine in the absence and presence of PAAS.

Table 5 and Fig. 14 present the binding energies of elements on the serpentine surface in the absence and presence of PAAS, This result was consistent with the previous research results^6,43^. After serpentine was treated by PAAS, the chemical shift of Si was not obvious, while that of Mg shifted + 0.1 eV, which indicated the change of the chemical environment. It indicated that the carboxyl groups on PAAS chemically react with Mg on the serpentine surface. This was consistent with the zeta potential and infrared spectral (IR) analysis results^15^.

**Table 5:** Binding energies of elements on the serpentine surface.

Sample	Binding energy (eV)		Shift energy (eV)	
	Mg (1s)	Si (2p)	Mg (1s)	Si (2p)
Serpentine	1303.28	102.48	–	–
Serpentine + PAAS	1303.38	102.48	+ 0.1	–

### Turbidity test analysis

**Figure 15:**
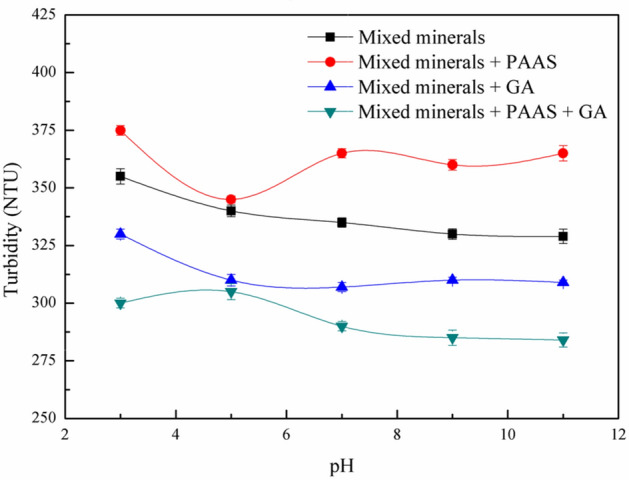
Relationship between dispersion of regulators on mixed minerals and pH (serpentine: talc = 1:1, mass ratio).

The surface synergism of PAAS and GA on the mixed minerals of serpentine and talc was studied by turbidity technique. The relationship between dispersion of regulators on mixed minerals and pH was shown in Fig. 15. In the pH range of 3–11, the turbidity of mixed minerals decreased with the increased of pH, indicating that there is a heteroaggregation between serpentine and talc, which is consistent with the result of zeta potential. From the Fig. 15, the turbidity of mixed minerals increased significantly when 24.7 mg/L PAAS was added, which indicated that PAAS can effectively disperse the mixed minerals of serpentine and talc. Different from PAAS, the turbidity of the mixed minerals decreased with the increase of pH when 100 mg/L was added, indicating that GA had a depression effect on the mixed minerals. Compared with single depressant, the turbidity of the mixed minerals decreased significantly when combined depressant consisting of PAAS and GA were introduced. Because the fine serpentine particles covering the surface of talc were dispersed by PAAS and it was conducive to the effective inhibition of talc by GA.

### Artificial mixed ore flotation

The surface synergism of PAAS and GA on the chalcopyrite and magnesium silicate minerals was verified through flotation separation operation of artificial mixed ore. The artificial mixed ore is composed of serpentine, talc and chalcopyrite (mass ratio 1:1:2). It can be seen from Table 6 that PAAS or GA alone could not effectively separate chalcopyrite from magnesium silicate minerals. Compared with single depressant, combined depressant consisting of PAAS and GA could simultaneously depress serpentine and talc. Finally, chalcopyrite and magnesium silicate minerals were separated effectively.

**Table 6:** Results of different depressants on flotation performance of artificial mixed ore.

Flotation condition	Grade of Cu (%)	Cu recovery (%)	Grade of MgO (%)	MgO recovery (%)
Mixed ore + PAAS (24.7 mg/L)	30.97	67.56	8.90	18.57
Mixed ore + GA (100 mg/L)	31.43	65.86	7.89	21.14
Mixed ore + PAAS (24.7 mg/L) + GA (100 mg/L)	46.62	90.45	1.43	4.61

### Mechanism of surface synergism between PAAS and GA

When serpentine and talc coexist in an ore, the surface charges of serpentine and talc are opposite in a wide pH range, and heteroaggregation easily occurs. The surface of talc is covered by fine serpentine particles due to heteroaggregation, which results in a significant decrease in the flotation recovery rate of the mixed ore. At the same time, fine serpentine particles cover the talc surface to form a dense film to prevent the adsorption of depressant (GA) on the surface of talc. Theoretical analysis of sodium polyacrylate (PAAS) and gum Arabic (GA) on flotation separation of chalcopyrite and magnesium silicate minerals were investigated above by zeta potential, infrared spectral (IR), SEM–EDS and XPS analysis.

Sodium polyacrylate (PAAS) is a negative polymer, its negatively charged carboxyl group (–COO–) is connected with the main chain. When pH > 9, the carboxyl group (–COO–) on sodium polyacrylate is completely dissociated and the molecular chain is extended. A large number of carboxyl groups (–COO–) chemically react with (Mg^2+^) on the serpentine surface to form chelating products. The chemical reaction consumes a large amount of Mg^2+^ on the surface of serpentine, the positive charge density of the site ions in the serpentine double layer is significantly reduced, which makes the zeta potential on the serpentine surface shift negatively. At this time, the electrostatic attraction between serpentine and talc is changed to electrostatic repulsion, and the heteroaggregation between serpentine and talc is broken^15,44^. The floatation recovery of talc is rapidly restored as the serpentine is dispersed, which explains the experimental results in Fig. 5. It is necessary to add another polymer depressant (GA) to depress talc. Gum Arabic (GA) is a kind of polysaccharide, based on the above discussion, the adsorption of GA on talc surface is mainly via physical interaction. In addition, hydrogen bonds may also play a positive role. GA is adsorbed on the surface of talc by hydrogen bonding, which makes the surface of talc more hydrophilic and the talc is depressed^28^.To the end, it is necessary to depress talc and serpentine simultaneously.

**Figure 16:**
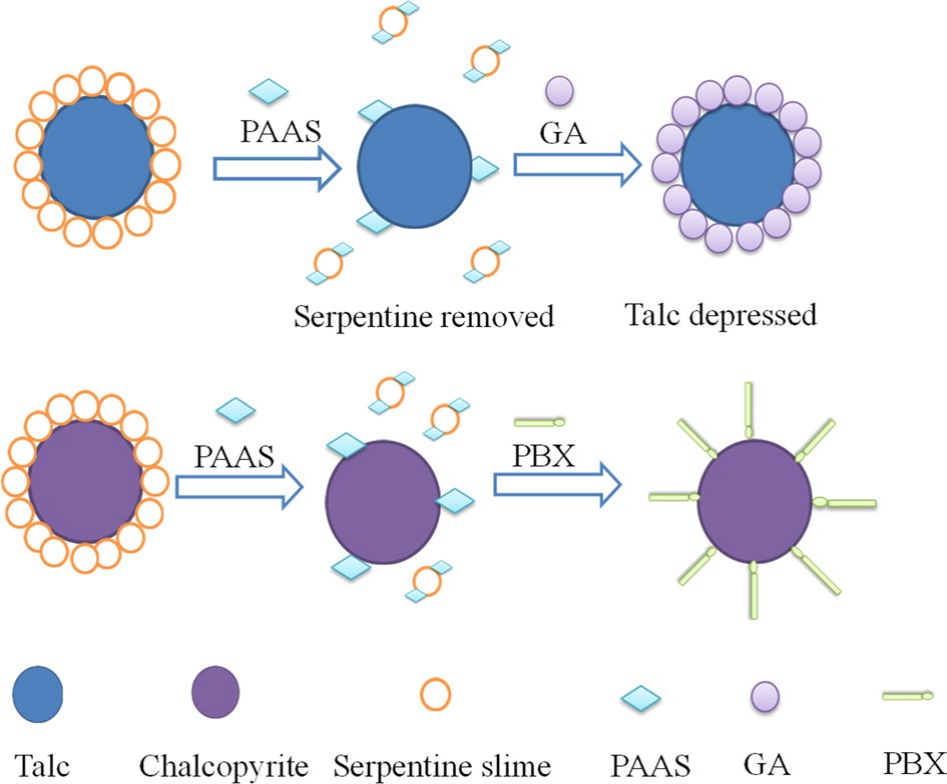
Depression mechanism of PAAS and GA.

In this study, combined depressant consisting of PAAS and GA are used to achieve simultaneous depression effect of talc and serpentine, and finally the copper-nickel mixed concentrate with copper grade of 4.43% and recovery of 88.18% is obtained. The depression mechanism is proposed and shown in Fig. 16. The surface of talc is covered by fine serpentine particles to form a dense film due to heteroaggregation, serpentine and talc particles produce a strong electrical repulsion when dispersant PAAS is added, which make serpentine and talc separate, and GA adsorbs on the surface of talc to depress its flotation, thus the adverse effect of talc on chalcopyrite flotation recovery is eliminated. Similarly, the serpentine adsorbed on the surface of chalcopyrite is removed by the dispersant PAAS, and finally the chalcopyrite obtained a better flotability using PBX as collector.

### Results of copper sulphide ore beneficiation test

The flotation results of copper sulfide ore are shown in Table 7. At pH 9.2, PAAS and GA were used as combined depressant and PBX as collector to form a closed cycle mixed flotation separation test. It can be seen from Table 6 that the copper-nickel mixed concentrate with copper grade of 4.43% and recovery of 88.18% was obtained by using PAAS and GA combined depressant. Compared with single depressant, the copper grade and recovery were significantly improved of copper-nickel mixed concentrate by using combined depressant. The results showed that combined depressant consisting of PAAS and GA had significant depression effect on magnesium silicate minerals in copper sulfide ore.

**Table 7:** Results of copper sulphide beneficiation at pH = 9.2.

Flotation condition	Product	Yield (%)	Grade of Cu (%)	Cu recovery (%)
PAAS 80 g/t	Copper-nickel mixed concentrate	5.79	3.70	66.95
	Tailing	94.21	0.11	33.05
	Feed	100	0.32	100
CMC 200 g/t	Copper-nickel mixed concentrate	5.23	3.53	59.55
	Tailing	94.77	0.13	40.45
	Feed	100	0.31	100
GA 300 g/t	Copper-nickel mixed concentrate	5.33	3.51	58.46
	Tailing	94.67	0.14	41.54
	Feed	100	0.32	100
PAAS 80 g/t + GA 300 g/t	Copper-nickel mixed concentrate	6.37	4.43	88.18
	Tailing	93.63	0.04	11.82
	Feed	100	0.32	100

## Conclusions

In this study, combined depressant consisting of PAAS and GA were used to achieve the simultaneous depression effect of serpentine and talc. According to the results of micro-flotation experiments, zeta potential measurement, infrared spectrum analysis, SEM–EDS, XPS analysis and turbidity test above, the following observations and conclusions can be reached as follows.

In the flotation test, the single depressant has little depression effect on serpentine and talc mixed minerals. Compared with single depressant, the application of combined depressant consisting of PAAS and GA can improve separation efficiency of chalcopyrite and magnesium silicate minerals. Through flotation of Copper-Nickel Sulfide Ores, the copper-nickel mixed concentrate with copper grade of 4.43% and recovery of 88.18% is obtained. In the pH range of 2–12, the surface of serpentine and talc particles are opposite to each other and prone to heteroaggregation, which makes fine serpentine particles cover the surface of talc, preventing the depression effect of GA on the surface of talc. The surface synergism and mechanism of PAAS and GA on the mixed minerals of serpentine and talc is proposed by turbidity technique. The addition of dispersant PAAS can effectively disperse fine serpentine particles on the surface of talc and eliminate heteroaggregation between serpentine and talc. It is observed that GA can depress the floatability of talc and reduce the adverse effects on the recovery of copper concentrate. The combination of PAAS and GA can depress serpentine and talc simultaneously, reduce the MgO content in the concentrate.

## References

[1] Yamamoto T, Wu Z, Zhang L (1988). Flotation of copper-nickel sulphide ores from the Jinchuan Mine, China (I): On the effects of pulp temperature, pH values and sodium hexametaphosphate. Bull. Res. Inst. Miner. Dress. Metall. Tohoku Univ..

[2] Bampole DL, Mulaba-Bafubiandi A-F (2020). Mesophilic bioleaching performance of copper, cobalt and nickel with emphasis on complex orebodies of the Democratic Republic of Congo: A review of dynamic interactions between solids loading, microbiota activity and growth. Energy Ecol. Environ..

[3] Marakushev AA, Paneyakh NA, Zotov IA, Lo ZH, Su SG (2000). The Dzhinchuan copper-nickel deposit in China, and the relation of the PGE mineralization to the ultrabasic rock alkalinity. Geol. Ore Deposits.

[4] Feng B (2018). Use of locust bean gum in flotation separation of chalcopyrite and talc. Miner. Eng..

[5] Martins J, Amarante M (2013). Scheelite flotation from Tarouca mine ores. Miner. Process. Extr. Metall. Rev..

[6] Tang X, Chen Y (2020). Using oxalic acid to eliminate the slime coatings of serpentine in pyrite flotation. Miner. Eng..

[7] Zhao K (2020). Effect of a novel phosphate on the flotation of serpentine-containing copper-nickel sulfide ore. Miner. Eng..

[8] Guo W, Feng B, Peng J, Zhang W, Zhu X (2019). Depressant behavior of tragacanth gum and its role in the flotation separation of chalcopyrite from talc. J. Mater. Res. Technol. Jmr&T.

[9] Long T, Xiao W, Yang W (2019). The effect of molecular assembly between collectors and inhibitors on the flotation of pyrite and talc. R. Soc. Open Sci..

[10] Zhang X, Han Y, Gao P, Gu X, Li Y (2020). An Investigation into the Effects of Grinding Media on Grinding Products Characteristics and Flotation Performance of Pyrite. Miner. Process. Extr. Metall. Rev..

[11] Li C, Farrokhpay S, Shi F, Runge K (2015). A novel approach to measure froth rheology in flotation. Miner. Eng..

[12] Likhacheva SV, Neradovskiy YN (2017). Talc and serpentine particles morphology effect upon their distribution in flotation products (through the example of the Pechenga copper-nickel ores). Obogashchenie rud.

[13] Feng B, Lu Y, Feng Q, Zhang M, Gu Y (2012). Talc-serpentine interactions and implications for talc depression. Miner. Eng..

[14] Yang S, Xie B, Lu Y, Li C (2018). Role of magnesium-bearing silicates in the flotation of pyrite in the presence of serpentine slimes. Powder Technol..

[15] Liu D, Zhang G, Huang G, Gao Y, Wang M (2019). The flotation separation of pyrite from serpentine using lemon yellow as selective depressant. Colloids Surf. A Physicochem. Eng. Asp..

[16] Lu J (2019). Innovative insight for sodium hexametaphosphate interaction with serpentine. Colloids Surf. A Physicochem. Eng. Asp..

[17] Castellon CI (2020). Depression of Pyrite in seawater flotation by Guar Gum. Metals.

[18] Pan G, Zhang G, Shi Q, Chen W (2019). The effect of sodium alginate on chlorite and serpentine in chalcopyrite flotation. Minerals.

[19] Feng B (2019). The flotation separation of galena and pyrite using serpentine as depressant. Powder Technol..

[20] Zhao K (2015). The effect of a new polysaccharide on the depression of talc and the flotation of a nickel-copper sulfide ore. Miner. Eng..

[21] Zhao K (2016). The effect of a new polysaccharide on the depression of talc and the flotation of a nickel-copper sulfide ore (vol 77, pg 99, 2015). Miner. Eng..

[22] Kruger M, Naik S, Naudé N (2018). Upgrade of SLon concentrate with the use of froth flotation on a typical African iron ore. Miner. Process. Extr. Metall. Rev..

[23] Hu H, Li M, Li L, Tao X (2020). Improving bubble-particle attachment during the flotation of low rank coal by surface modification. Int. J. Min. Sci. Technol..

[24] Chen Y, Zhang G, Shi Q, Yang S, Liu D (2020). Utilization of tetrasodium iminodisuccinate to eliminate the adverse effect of serpentine on the flotation of pyrite. Miner. Eng..

[25] Feng B (2018). Synergistic effect of acidified water glass and locust bean gum in the flotation of a refractory copper sulfide ore. J. Clean. Prod..

[26] Liao R, Feng Q, Wen S, Liu J (2020). Flotation separation of molybdenite from chalcopyrite using ferrate(VI) as selective depressant in the absence of a collector. Miner. Eng..

[27] Zhao K (2017). Dispersive effect of low molecular weight sodium polyacrylate on pyrite-serpentine flotation system. Physicochem. Probl. Miner. Process..

[28] Liu D, Zhang G, Huang G, Gao Y, Wang M (2019). Investigations on the selective flotation of chalcopyrite from talc using gum Arabic as depressant. Sep. Sci. Technol..

[29] Wang W (2018). Comparative study on adsorption and depressant effects of carboxymethyl cellulose and sodium silicate in flotation. J. Mol. Liq..

[30] Jiang H (2018). Adsorption behaviors and mechanisms of dodecyltrimethyl ammonium chloride and cetyltrimethyl ammonium chloride on illite flotation. Powder Technol..

[31] Zhang X (2020). The flotation separation of molybdenite from chalcopyrite using a polymer depressant and insights to its adsorption mechanism. Chem. Eng. J..

[32] Yang B (2020). A novel copper depressant for selective flotation of chalcopyrite and molybdenite. Miner. Eng..

[33] Qin W (2013). Utilization of polysaccharides as depressants for the flotation separation of copper/lead concentrate. Int. J. Mining Sci. Technol..

[34] Liu C, Zhang W, Song S, Li H (2019). A novel method to improve carboxymethyl cellulose performance in the flotation of talc. Miner. Eng..

[35] Pan G, Shi Q, Zhang G, Huang G (2020). Selective depression of talc in chalcopyrite flotation by xanthan gum: Flotation response and adsorption mechanism. Colloids Surf. A.

[36] Liu D, Zhang G, Huang G, Gao Y, Wang M (2019). Investigations on the selective flotation of chalcopyrite from talc using gum Arabic as depressant. Sep. Sci. Technol..

[37] Li H, Tripp CP (2004). Interaction of sodium polyacrylate adsorbed on TiO_2_ with cationic and anionic surfactants. Langmuir.

[38] Li J (2018). Biological macromolecule delivery system fabricated using zein and gum arabic to control the release rate of encapsulated tocopherol during in vitro digestion. Food Res. Int..

[39] Bai L, Liu J, Han Y, Jiang K, Zhao W (2018). Effects of xanthate on flotation kinetics of chalcopyrite and talc. Minerals.

[40] Pan G, Shi Q, Zhang G, Huang G (2020). Selective depression of talc in chalcopyrite flotation by xanthan gum: Flotation response and adsorption mechanism. Colloids Surf. A Physicochem. Eng. Asp..

[41] Yuan D, Cadien K, Liu Q, Zeng H (2019). Separation of talc and molybdenite: Challenges and opportunities. Miner. Eng..

[42] Ulrich M, Muñoz M, Guillot S, Cathelineau M, Couteau C (2014). Dissolution-precipitation processes governing the carbonation and silicification of the serpentine sole of the New Caledonia ophiolite. Contrib. Miner. Petrol..

[43] Cao C-Y, Yu B, Wang M, Zhao Y-Y, Zhao Y-H (2019). Adsorption properties of Pb2+ on thermal-activated serpentine. Sep. Sci. Technol..

[44] Qin W (2012). Effect of sodium pyrophosphate on the flotation separation of chalcopyrite from galena. Int. J. Min. Sci. Technol..

